# Multiple Gastric Metastases from Ovarian Carcinoma Diagnosed by Endoscopic Ultrasound with Fine Needle Aspiration

**DOI:** 10.1155/2012/610527

**Published:** 2012-07-01

**Authors:** Mehmet Akce, Sharon Bihlmeyer, Andrew Catanzaro

**Affiliations:** ^1^Department of Internal Medicine, Saint Joseph Mercy Hospital, Ann Arbor, MI 48106, USA; ^2^Department of Pathology, Saint Joseph Mercy Hospital, Ann Arbor, MI 48106, USA; ^3^Department of Gastroenterology and Internal Medicine, Saint Joseph Mercy Hospital, Ann Arbor, MI 48106, USA

## Abstract

Metastasis to the stomach from nongastric tumors is a rare event. We present a case of ovarian cancer metastasis to the gastric wall that presented as multiple subepithelial gastric lesions. A 55-year-old female with known stage III b serous ovarian cancer was admitted to the hospital with melena and anemia. A 1.5 to 2 cm subepithelial mass with superficial overlying erosion in the antrum was seen in Esophagogastroduodenoscopy (EGD). Initial endoscopic mucosal biopsies were normal. An Endoscopic Ultrasound (EUS) was performed, which revealed two subepithelial lesions with the typical appearance of a gastrointestinal stromal tumor. Fine needle aspiration (FNA) of both masses revealed papillary adenocarcinoma from an ovarian papillary serous adenocarcinoma. This is the first reported case of multiple gastric metastatic lesions from ovarian cancer diagnosed by EUS FNA.

## 1. Introduction 

Metastasis to the stomach is uncommon. Ovarian tumors comprise 0.013% to 1.6% of all gastric metastatic tumors [1, 2]. Gastrointestinal involvement from these tumors is often mucosal and associated with ulceration [3]. We present a case of ovarian cancer metastasis to the gastric wall, which presented as multiple subepithelial gastric lesions. This was diagnosed by endoscopic ultrasound with fine needle aspiration (EUS-FNA).

## 2. Case Presentation 

**Figure 1:**
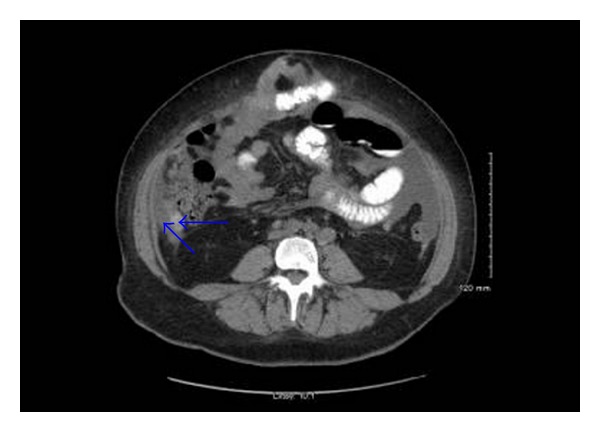
CT Abdomen without IV contrast, revealing calcified, heterogeneous, mixed intermediate, and high-density deposits worrisome for peritoneal carcinomatosis.

**Figure 2:**
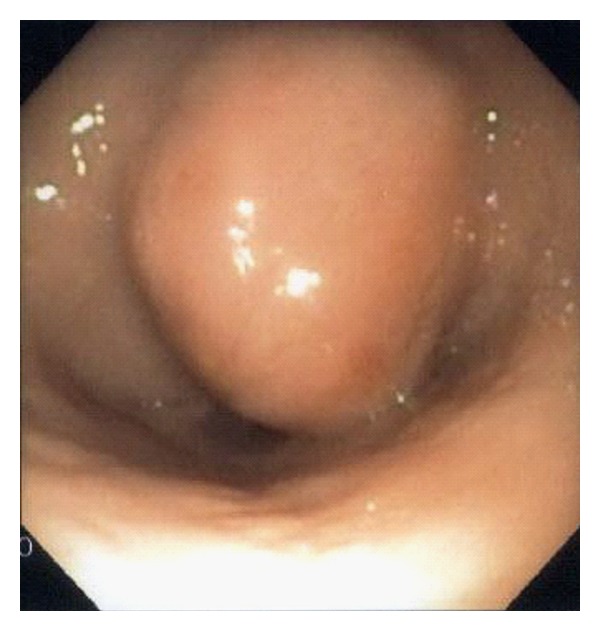
Subepithelial round mass in the antrum measuring from 1.5 to 2 cm, resembling gastrointestinal stromal tumor.

**Figure 3:**
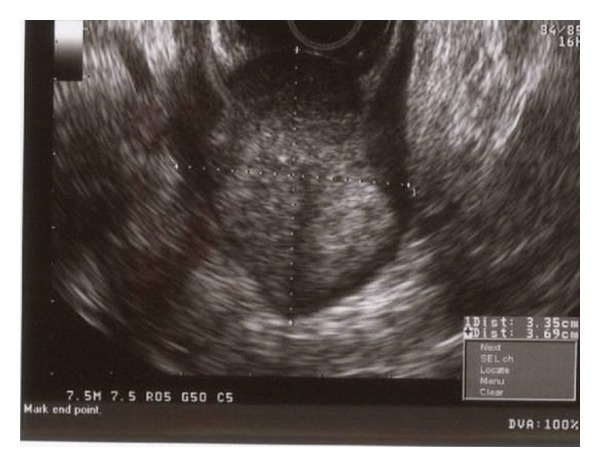
EUS image of antral mass measuring 3.4 × 3.7 cm. Hypoechoic mass emanating from the muscularis propria and with the typical appearance of gastrointestinal stromal tumors.

**Figure 4:**
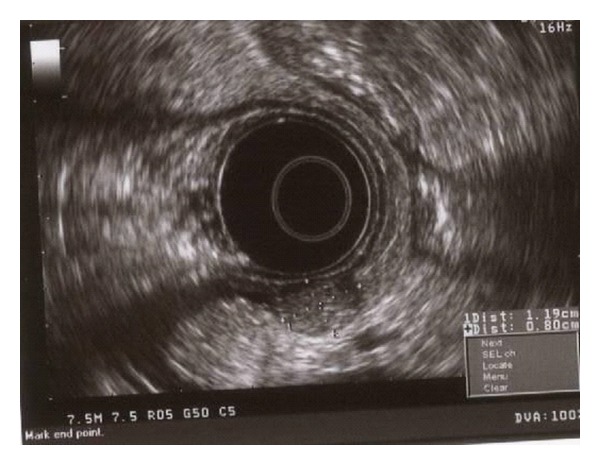
EUS picture of the subepithelial mass measuring 1.2 × 0.8 cm in the body of the stomach. Hypoechoic mass emanating from the muscularis propria and with the typical appearance of gastrointestinal stromal tumors.

**Figure 5:**
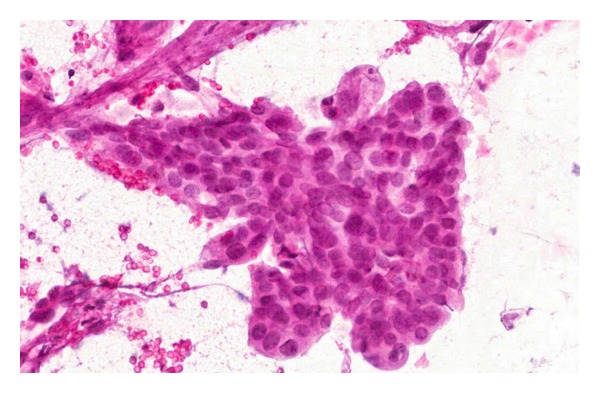
Microscopic image from FNA specimen. 40x—FNA prepared slide (smeared) at high power (Pap stain) showing a group of cells in a papillary arrangement with rounded borders. The cells have round to oval nuclei with slight pleomorphism and variably prominent nucleoli.

The patient is a 55-year-old female with known stage III b serous ovarian cancer. She had undergone an abdominal hysterectomy and bilateral salpingo-oophorectomy with omentectomy, followed by 6 cycles of carbo/taxol chemotherapy with complete clinical response. She was free of disease for 2 years until her disease recurred and was treated with Carboplatin and Taxol. The carboplatin was eventually switched to Doxil. However, the repeat positron emission tomography (PET) scan at that time showed progression of her disease. Thus she underwent exploratory laparotomy with removal of a splenic mass. She was noted to have peritoneal carcinomatosis at that time and was then treated with Gemzar. The patient had stable disease after this treatment. Five years after the initial diagnosis, the patient was admitted to the hospital with anemia, hemoglobin of 7.0 gm/dl, fatigue, and melena. Computerized Tomography (CT) of the abdomen without IV contrast was obtained on admission, which revealed calcified, heterogeneous, mixed intermediate and high-density deposits worrisome for peritoneal carcinomatosis (Figure 1, arrows). No IV contrast was administered due to her poor kidney function. She was referred for an EGD, which showed a 7 mm erythematous lesion at the gastroesophageal junction and a 1.5 to 2 cm subepithelial mass (Figure 2) with a superficial overlying erosion in the antrum, but no obvious source for any active bleeding. Initial endoscopic biopsies of the gastroesophageal junction lesion showed granulation tissue polyp with foveolar hyperplasia, and the antral biopsies were normal. Due to the presence of a subepithelial lesion in the antrum, the patient was referred for EUS. Two subepithelial lesions were discovered by EUS, one in the antrum measuring 3.4 × 3.7 cm (Figure 3) and one in the body of the stomach 1.2 × 0.8 cm (Figure 4). The lesion in the body of the stomach was not appreciated during the EGD. The lesions were hypoechoic masses emanating from the muscularis propria and had the typical appearance of gastrointestinal stromal tumors. FNA was performed of both masses. Both sites revealed papillary adenocarcinoma from an ovarian papillary serous adenocarcinoma primary (Figure 5). Immunostains for progesterone receptor, estrogen receptor and p 53 were focally positive and confirmatory. The patient was treated with Taxol and is undergoing surveillance imaging.

## 3. Discussion 

The tumors most commonly reported to metastasize to the stomach include melanoma, breast, lung, and esophageal carcinoma [1, 2, 4, 5]. Clinical manifestations of metastasis to stomach are variable and include epigastric pain, melena, anemia from occult gastrointestinal blood loss, nausea, and vomiting [1–3, 6, 7]. Ulcerated nodules, ulcerated submucosal masses, umbliciated nodules with central exudate, and necrotic ulcers with heaped up margins were reported to be the most common endoscopic findings by Kadakia et al. [8]. The gastric metastasis can be solitary (62.5%–65%) or multiple (35%–37.5%) and more commonly located in the middle or upper third of the stomach [2, 9]. 

Ovarian tumor metastasis to the stomach is uncommon [1, 2]. Ovarian carcinoma is usually confined to the peritoneal cavity at presentation and throughout its course in approximately 85% of patients [3]. It regularly metastasizes to peritoneal surfaces by exfoliating cells that implant throughout the peritoneum and the intraperitoneal route of dissemination is considered the most common [3, 10, 11]. Gastrointestinal involvement is usually limited to seromuscular layer of the small and large bowel and its mesentery [12]. However, it may also metastasize through the lymphatic channels and hematogenous route [11]. Based on the presence of peritoneal carcinomatosis, intraperitoneal route of dissemination of the ovarian carcinoma to gastric wall may be possible in our case. However, hematogenous spread cannot be ruled out in the presence of the well-circumscribed lesions in the gastric wall without adjacent intraperitoeal mass. Gastrointestinal involvement is most often superficial, and transmural invasion is less common [9]. Even though it may present as a gastric metastasis at advanced stages there have been some reports in the literature describing gastric metastasis as an initial presentation of ovarian cancer [2, 3].

Similar to our case, there have been other reported cases of metastatic ovarian cancer presenting as single subepithelial gastric lesions. The diagnoses in these cases were made by surgical exploration and endoscopic submucosal dissection [3, 13]. There have been two cases reported in the literature where EUS-FNA was utilized for diagnosis of single gastric metastasis from ovarian carcinoma [14, 15]. Alternatively, we present a case of multiple gastric metastatic lesions from ovarian carcinoma diagnosed by EUS FNA. The fine needle aspiration was imperative in the diagnosis of this patient as the lesions appeared by endoscopic ultrasound to be gastrointestinal stromal tumors given their location and ultrasound appearance.

## 4. Conclusion

Metastatic disease should be in the differential diagnosis of the patient presenting with subepithelial gastric lesions. Endoscopic ultrasound with fine needle aspiration is invaluable for making the correct diagnosis of gastric subepithelial lesions and should be considered in all cases if available.
